# 2019 Asian American/Pacific Islander Nurses Association & Taiwan Nurses Association Joint International Conference: Changes in Nursing Research, Education, and Practice: From Local to Global

**Published:** 2020

**Authors:** Young-Shin Lee, PhD, RN

**Affiliations:** 2019 Conference Planning Chair

The 2019 Conference Planning Committee Chair’s Message:

The 2019 Asian American/Pacific Islander Nurses Association (AAPINA) & Taiwan Nurses Association (TWNA) Joint International Conference was held with the theme of ***Changes in Nursing Research, Education, and Practice: From Local to Global*** on August 16–17, 2019 at the Splendor Hotel, Taichung City, Taiwan. More than 700 researcchers, educators, and clinical nurses from over 10 countries participated in the conference. A dozen of internationally well-known nursing scholars and leaders including Dr. Pamela F. Cipriono, the Vice President of International Councl of Nurses (ICN), Dr. Wen-Ying Chou, the Program Director of Health Communication and Informatics Research Branch, the National Cancer Institue of the USA, and Dr. Eun-Ok Im, the AAPINA Presidetn-Elect, and Dr. Hsiu-Hung Wang, the TWNA President**provided keynote speeches. In addition, seven international scholars and leaders provided a thought-provoking forum on changes in nursing leadership in Asian countries. 

In the AAPINA section, a total of 80 abstracts were presented. The main topics of the presentations were: (a) Clinical - Care management and effect; (b) Challenges in Care: Nurse Burn Out, Caregiver Stress, and Folk Medicine; (c) Youth Perspectives toward Health; (d) Leadership in Nursing; (e) Trajectory of Cancer - What we know about; (f) Research in Clinical Environments; (g) End-of-Life Care; (h) Education in Nursing and the Evidence; (i) Nursing Working Force; and (j) Researh with Advanced Methods. The sympsia included: (a) the HEARTS of older persons in Pittsburgh, PA and the Philippines - A global collaboration; (b) the Effects of Technology-Based Interventions on Health Outcomes; and (c) Assessing the HEARTS of Older Persons in Mandaluyong, Philippines. In addition, the AAPINA provided an Editor’s forum on effective strategies for writing a publishable research manuscript during a luncheon session.

In short, we had an outstanding and highly productive conference with various sessions, symposia, and forums. The published abstracts were selected among highly competitive abstracts that were submitted to the conference, based on the result of peer reviews and the authors’ agreement. Please enjoy the abstracts that are presented in this issue. 

We are proudly present outstanding examples of our products.

**Abstract 1**

*Knowledge of Human Papillomavirus (HPV) Infection and HPV Vaccines in College Students*

**Bulaporn Natipagon-Shah, PhD**

California State University San Marcos, San Marcos, CA, USA 

**Background and Purpose:** Human Papillomavirus (HPV) is the most common sexually transmitted disease in the US. Some types of HPV causes cervical and throat cancer. The success of disease and cancer prevention mainly depends on the level of awareness and knowledge about the disease and the vaccine. Although the vaccination is recommended at aged 11-12, the CDC suggests that the vaccination can also be administered to those who had not been properly vaccinated at age before 26. The purpose of this study is to assess knowledge of HPV infection and vaccination, as well as the intention of receiving the vaccine among college students.

**Methodology:** This study employed a cross sectional descriptive quantitative design. The data was collected from 250 college students aged 19-25 who are voluntarily to complete about 20-minutes online self-administered anonymous questionnaire. Data was analyzed to identify the students’ level of knowledge of HPV and HPV vaccine, as well as their intention of receiving the vaccine in the next 6 months.

**Results:** A total of 250 students participated in this study. The average age of them are 23.5 years old, and 78% are females, 21.5% are males, and about 0.5 % are transgender. About 66% do not know whether the HPV vaccine offers protection against genital warts, about 57% do not know whether the HPV vaccine offers protection against most cervical cancers, about 53% do not know that the HPV vaccine requires at least 2 doses, and about 55% do not know that the HPV vaccine is recommended by Centers for Disease Control and Prevention (CDC) for females aged up to 26 who were not adequately vaccinated properly. Regarding the knowledge of the HPV, about 65% do not know that HPV infection can cause oral, penile, and/or cervical cancer. About 65% of the students do not know that men can get HPV infection, about 61% responded that HPV infection is very rare, and about 53% responded that HPV infection can be transmitted only when the person has symptoms. However, about 78% of the students stated the intention to receive the vaccine in the next 6 months.

**Conclusion and Recommendation:** More than half of the students in this study do not have common knowledge about the HPV vaccines and HPV infection. An intervention to increase awareness of HPV infection, and the important of receiving the HPV vaccine is needed for the collage-aged students. 

**Implications for Nursing:** More than half of the students in this study do not have common knowledge about the HPV vaccines and HPV infection. An intervention to increase awareness of HPV infection, and the important of receiving the HPV vaccine is needed for the collage-aged students. A proposal for free vaccines from a major drug company can increase the vaccination intake of the college students who do not have insurance and cannot afford to pay for the vaccine.

**Abstract 2**

*Students’ Living Experience and Communication with Older Adults and Their Attitudes to-ward Older People*

**Young-Shin Lee, PhD, RN**

San Diego State University, San Diego, CA, USA 

**Background:** Culture influences people’s lifestyle, perception, attitudes, and behavior. Modern society becomes individualized and atomizes the structure and function of family, which changes traditional lifestyle and culture. While fast-growing aging populations increase demands on health care providers, norms and perceptions toward aging have been changing which impacts the quality of care for older adults. Studies show that attitudes of young adults differ by gender, ethnicity and culture, and living experiences. 

**Purpose:** To compare the similarities and differences of attitudes and bias toward aging among nursing students within two different cultural backgrounds – Asian and Caucasian by demographics, level of contact and confidence of communication with older adults. 

**Methods:** A total of 308 students in a senior level of nursing program completed the Measures of Attitudes toward Older People and Aging Quiz online at the beginning of Gerontology course. ANOVA and 

Multiple Regression methods were applied to yield the goals of the study.

**Results:** Compared with the Caucasian students, more Asian students had a significant amount of living with older adults but reported less comfort communicating with them. Among positive and negative attitudes and pro- and anti-aged bias, Asian students indicated more negative and more anti-aged bias than Caucasian students, while positive attitudes and pro-aged bias did not have differences in the two groups. Multiple regression analysis showed that confidence of communication with older adults significantly influenced all variables of positive, negative, pro-aged, and anti-aged bias; Asians who were younger, had less time to communicate and were uneasy communicating had more negative attitudes, while those who lived together with older adults showed more Pro-aged bias. 

**Conclusions:** Developing students’ competency in communication with older adults is important to have positive attitudes. Frequent communication is a key to understanding older people and reducing nursing students’ negative attitudes and anti-aged bias.

**Implications for Nursing:** Nursing programs should provide increased opportunities for nursing students to communicate with older adults in both acute and community settings. 

**Abstract 3**

*Comparing The Effects Of Adolescent–Only And Parent–Teen Sexual Education Programs On Sexual Abstinence Intention Among Early Adolescents*

**Monrudee Chokprajakchad, MSN^1^, Rutja Phuphaibul, Prof.Dr.^1^, Renee E. Sieving, Prof.Dr.^2^, Srisamorn Phumonsakula, Assoc.Ph.D.^3^, Srisamorn Phumonsakula, Assoc. Ph.D.^1^**

^1^School of Nursing, Faculty of Medicine Ramathibodi Hospital, Rachtawi, BKK, Thail 

^2^School of Nursing, University of Minnesota, Minneapolis, USA

**Introduction/purpose:** Early sexual initiation is considered a serious problem worldwide. Meanwhile, the average age of first sexual intercourse among adolescents is declining. This experimental study design based on Ajzen’s Theory of Planned Behavior aimed to compare the effects of parent participation in an adolescent sexual education program on parent’s sexual communication behavior and adolescent’s sexual abstinence intention. 

**Methods:** Eighty seventh-grade students and their parents were randomly assigned to the adolescent only program (group A, n = 39) and parent-teen program (group B, n = 41). The descriptive statistics were used for demographic data analysis. Repeated measure ANOVA and Generalized Estimating Equations (GEE) were used to test the effects of the program.

**Results:** The findings at immediate and one month after the program indicated that sexual communication behavior scores of parents in Group B increased significantly over time (p < .05). Additionally, the attitude, norms, and intention about sexual communication of parents in Group B significantly increased over time and were higher than those in Group A (p < .05). Students in Group B showed a higher score of norms about sexual abstinence compared to those in Group A immediately after completing the intervention (B=12.93, p < .001). However, the attitude, perceived behavior control, and intention of sexual abstinence scores of students between Groups A and B were not significantly different across the time points of measurements. The finding suggested that the involvement of both parents and adolescents into sex education and sexual communication program would be more effective than the adolescent-only program. Future research should be involved parent in a program and designed to gain more confidence and skill about parents’ sexual communication.

**Discussion:** The finding indicated the parent–based expansion of the TPB to include parenting influences explicitly and indicated a conceptual framework for family-based design to increase sexual communication between parent and their adolescent child also promote adolescent’ perceptions and sexual abstinence intention. In addition, behavioral belief and normative belief were also found to have the highest effect on parent sexual communication. While perceiving norms about sexual abstinence intention was also found to have the highest effect on adolescents among the other constructs from the TPB in this study. Future research should be involved parent in a program and designed to gain more confidence and skill about parents’ sexual communication. Moreover, the programs can be applied as guidelines for nurses who work in schools, communities, and adolescent health services in health promotion. Nurses should push for the use of the intervention in schools and communities with the cooperation of parent-teacher association.

**Abstract 4**

*Constructing the evaluation tool of teaching quality for nurse clinical teachers in a medical center*

**Shirling Lin, MSN, RN**

Taipei Veterans General Hospital, Taipei, Taiwan, Taiwan 

**Background:** By using the appropriate teaching designs and methods, that learners could learn knowledge, morality and behavior comprehensively. Assessment and improvement of these specific competencies require an instrument measuring teacher performance. 

**Objective:** The purpose of this study was to develop and validate of a questionnaire to assess the nurse clinical teachers’ competencies essential for facilitating reflective learning. 

**Methods:**A cross-sectional study design was used and data were collected by questionnaires. A stratified sampling of totally 39 classes including 752 staff nurses was invited from a medical center via anonymous intranet data collecting. Research instruments were used the 25-item teaching evaluation tool. ANOVA, t-test, factor analysis, cluster analysis, and discriminant analysis were performed for data analysis. 

**Results:** The findings were the following: (1) The score of “the content of the lecture is fully prepared and practical” was higher, the score of “clear understanding of the students’ prior knowledge and experiences” was lower; (2) The N4-level classes had higher composite teaching evaluation scores; (3) Five factors were extracted and named from the teaching evaluation tool, including course content, teacher-student interaction, teaching method, overall evaluation, and student self-assessment. These factors accounted for 84.4% of the total variance; (4) Knowledgeable type and interactive type was derived from cluster analysis from the teaching evaluation tool and the distinguished rate was 98.8% by discriminant analysis.

**Conclusions:** These results were to provide administrators with actionable recommendations that can be used to develop a practical tool, to value learners’ perceptions of their teachers’ competencies for facilitating learning. Furthermore, it would be referred to promote the instrument and enhance the teaching quality and educational effectiveness.

**Implications for Nursing:** It would be referred to promote the instrument and enhance the nursing teaching quality and educational effectiveness.

**Abstract 5**

*Learning Experience and the effects of the Global Nursing Partnership Program*

**Hyang-yon Rhee, PhD^1^, Suhyun Bae, Ed.M^1^, Sook Ja Yang, PhD^1^, Sung-Heui Bae, PhD^2^, Somana Svay, M.P.H.M^2^**

^1^Ewha Womans University, Seoul, S. Korea

^2^Technical School for Medical Care, Phnom Penh, Cambodia

**Background:** In 2008, the Cambodian government announced Health Strategic Plan to train high-quality nurses, however by 2015, only 5 percent of nurses had a bachelor’s degree. In this study, our research group operated the Nurse Bridge Program (NBP) from July 2017 to December 2018 in cooperation with the University of Health Sciences (UHS) in Cambodia under the support of Korea International Cooperation Agency (KOICA). The objective of the NBP was to improve nursing and research competencies of students with an associate degree in nursing and to award the bachelor’s degrees. The NBP adopted team teaching with UHS faculties to advance their teaching and research competencies.

**Purpose:** The purpose of the study is to explore the changes before and after the program in nursing and research competencies of students (i.e. program participants) as well as in teaching and research competencies of UHS nursing faculty members.

**Methods:** Our research participants were forty-two students and 21 faculty members. We utilized survey tools from Cowanan (2008), Pager (2012), and Hwang (2006) to measure participants’ nursing, research, and teaching competencies, respectively. Data were collected through three surveys (pre-test, mid-test, post-test) in July 2017, February and December 2018. Survey data were analyzed using descriptive statistics, t-test, and repeated measures ANOVA. We further conducted focus group interviews (FGI) after the completion of the curriculum to identify the qualitative effects of the NBP based on the participants’ experiences.

**Results:** Students’ nursing competencies-care delivery and assessment skills, were gradually increased through the pre-, mid-, and post-test and there was a significant difference in assessment skill between the pre- and the post-test. There was also a significant difference in students’ research competency between the mid-and the post-test. Regarding the teaching and research competencies of faculties, there was no significant difference across surveys. The detailed learning experience of students and faculties and effects of the program were reported through FGI.

**Conclusions:** The NBP as a global nursing partnership program not only enhances the professional competencies of nursing students and faculties but also provides a meaningful learning experience. Ongoing global partnerships and support are expected to foster professional high-quality nurses and contribute to the improvement of health care in Cambodia.

**Implications for nursing** is to improve professional nursing competencies and contribute to the improvement of health care in Cambodia.

**Abstract 6**

*A Situation-Specific Theory of Korean Nurses’ Role Transition Experience*

**You Lee Yang, PhD, RN^1^, Eun-Ok Im, PhD, MPH, RN, CNS, FAAN^1^**

^1^Duke University School of Nursing, Durham, USA

**Aims:** Numerous empirical studies have been conducted on nurses’ role transition. However, there is no adequate theory of Korean nurses’ role transition experience, especially in situations of South Korea where there is no clinical ladder system. The purpose of this study was to develop a situation-specific theory (SST) that explains the role transition of South Korean nurses in acute care settings.

**Methods:** Using an integrative approach, three sources for theorizing were used to develop the SST: (a) the conceptual model of role transition for nurses (adopted from Meleis (2010)’s transitions theory; (b) a literature review using multiple databases including the Cumulative Index for Nursing and Allied Health Literature (CINAHL), PubMed, PsycINFO, and two Korean databases (KISS and RISS); and (c) research findings from a structural equation modeling study on role transition of Korean critical care nurses and its related concepts. 

**Results:** The proposed SST included three major concepts with several sub-concepts: 1) transition conditions (intrapersonal factors, interpersonal factors, work environmental factors, and cultural factors), 2) patterns of response (successes in role transition and professional socialization), and 3) nursing therapeutics (education and leadership). The SST explains the relationships among these major concepts and sub-concepts. 

**Conclusion:** The proposed SST is limited in scope to acute care settings. However, it could help understand Korean nurses’ role transitions in South Korean contexts and could guide nursing education and management for nurses’ successful role transitions in South Korea. Further studies are needed to validate the proposed theory in various nursing settings and situations. 

**Abstract 7**

Relationship between Recognition of Symptoms in Patients with Colorectal Cancer and Knowledge of Risk Factors and Lifestyle

**Hiromi Uchihara, MSN, BSN, RN^1^, Midori Kamizato, PhD, PHN, RN^1^**

^1^Okinawa Prefectural College of Nursing, Naha City, Japan

**Background:** Colorectal cancer has characteristic symptoms such as persistent bleeding, diarrhea, constipation, and more. On the other hand, in interviews with colorectal cancer patients, which were conducted before the start of this study, it was found that they did not recognize the symptoms of colorectal cancer, even after onset. Therefore, it is important to encourage patients to recognize the symptoms of colorectal cancer. 

**Purpose:** The purpose of this study was to clarify the relationship between the recognition of colorectal cancer symptoms in affected patients and the knowledge of risk factors and lifestyle.

**Method:** Postoperative patients with colorectal cancer participated in a self-report questionnaire survey on “recognition of symptoms”, “knowledge of risk factors”, and “current lifestyle habits.” (Pearson’s Chi-squared test and Kruskal-Wallis test were) performed on “recognition of symptoms”; each question had one of three possible answers: “I didn’t know symptoms”, “I knew symptoms after onset”, and “I knew symptoms before onset”. And, analysis was performed on the relationship among “recognition of symptoms”, “knowledge of risk factors”, and “current lifestyle habits”.

**Result:** Responses were received from ninety-five patients (response rate: 88%). Thirty-six patients (37.9%) who answered “I didn’t know symptoms” were male and Stage II/III patients with less than 3 million yen in annual income; their knowledge of risk factors was low, and they reported high alcohol and fat intake and low vegetable intake. Forty patients (42.1%) who answered “I knew symptoms after onset” were female and Stage II/III patients; they reported high intake of vegetables and low alcohol and fat intake. Nineteen patients (20.0%) who answered “I knew symptoms before onset” were Stage 0/I patients and more than 3 million yen in annual income; their knowledge of risk factors was high and their fat intake was low.

**Conclusion:** In spite of after onset, about forty percent patients still do not know their symptoms. Differences in the knowledge of risk factors and lifestyle were observed because of differences in the recognition of symptoms. It is important that nurse need to provide the patients for depending on their knowledge of symptom.

**Implications for Nursing:** It is important that nurse need to provide the patients for depending on their knowledge of symptom.

**Abstract 8**

*Relationship between late effects and social isolation after radiation therapy in head and neck cancer survivors*

**Tomoharu Genka, MSN, BSN, RN^1^, Midori Kamizato, PhD, RN, PHN^1^**

^1^Okinawa Prefectural College of Nursing, Yogi, Naha, Japan

**Background:** Head and neck cancer survivors after radiation therapy have characteristic of late effects. In particular, late effects related to communication disorders are expected to reduce social interaction with the surrounding environment and to induce social isolation. The psychosocial experience of head and neck cancer survivors is characterized by high depression prevalence, relationship conflict, social isolation and high suicide rates.

**Purpose:** The purpose of this study was to clarify the relationship between late effects after radiation therapy and social isolation in head and neck cancer survivors.

**Method:** A cross-sectional, correlation design was adopted. Head and neck cancer survivors more than 3 months after radiation therapy were selected. Late effects and QOL were measured by the European Organization for Research and Treatment of Cancer (EORTC) quality of life questionnaire core module (QLQ-C30) and head and neck cancer module (QLQ-H&N35). Social isolation was measured with the Japanese version of the University of California Los Angeles loneliness scale ver.3.0 (UCLA-LS). The analysis determined the correlation coefficient between each variable.

**Result:** One hundred and one people were included in the analysis. The mean age of the patients was 62.3(± 11.4) years, and the period from the end of radiation therapy was35.2 (± 41) months on average. The symptom with the highest prevalence was “Trouble with social eating “(87.1%). Among the late effect by the participants, the severity of “Dry mouth” was the strongest. The average score of the loneliness scale was 44.2 (± 10.2) point, and this score was a high value compared with healthy people of the same age. Of the 14 late adverse events, 11 symptoms were correlated with social loneliness scores. Eleven symptoms were “Speech problems “(p < . 00, r < . 40), “Trouble with social contact” (p < .00, r < −.45) ,”Felt ill” (p < .00, r < . 37) “cough” (p < .00, r < .035) was included. Social isolation scores were correlated with overall QOL scores. In addition, functional QOL domain that was correlated with social isolation score was “emotional function” (p < 00, r <  -.049), “social function” (p < 00, r <  -.045), “cognitive function” (p > 00, r < −.039), and “Role function” (p > 00, r < −. 030).

**Conclusion:** Late effects in head and neck cancer survivors after radiation therapy may increase social loneliness. Nurses need to assess not only symptom management but also the psychosocial experience for survivors.

**Implications for Nursing:** Nurses need to assess not only symptom management but also the psychosocial experience for survivors.

**Abstract 9**

*Defecation Control with Fermented Food and Lactulose in Residents of a Long-Term Care Facility in Japan*

**Hiromi Hirata, PhD^1^, Hiromi Yamada, MSN^2^**

^1^Nihon Fukushi University, Tokai, Aichi, Japan

^2^Tsubutsubu Zakkoku Cooking Hidamari, Hikone, Japan

**Background:** Older people are prone to constipation due to decreased abdominal pressure, disturbed defecation reflexes, and weaker anal sphincter (Kitamura et al., 2016). Constipation is associated with many issues, including hypermagnesemia induced by long-term laxative use. 

**Purpose:** This study aimed to examine the effectiveness of non-laxatives such as fermented food (amazake) and lactulose (disaccharide) formulations for treating constipation in residents of a long-term care facility in Japan.

**Methods:**Eight residents who had been taking magnesium oxide as a laxative were recruited. The residents started drinking roughly 100 ml of amazake, a sweet non-alcoholic fermented rice drink, for two weeks after discontinuing the use of magnesium oxide. Then, after a two-day interval, they started drinking roughly 10 - 30 ml of lactulose for two weeks. The number of bowel movements, amount and property of stools, and use of enemas and rectal laxatives were examined. 

**Results:** Participants were one male and seven females at a mean age of 93 years. Four participants (B, D, E, and H) were able to sit on a toilet and exert pressure on the abdomen, one (A) was able to sit on a toilet but not exert pressure on the abdomen, and three (C, F, and G) used diapers. During the intervention, three participants (B, E, and H) had spontaneous defecation most of the time, whereas four (A, C, D, and F) showed no changes with either amazake or lactulose. One participant (G) who showed no change with amazake had spontaneous defecation twice while taking lactulose. 

**Conclusion:** Amazake and lactulose were not effective for treating constipation in participants who were unable to sit on a toilet and exert pressure on the abdomen, except for one participant who had spontaneous defecation while taking lactulose.

**Implications for Nursing:** These findings may be used for residents in nursing homes to treat constipation.

**Abstract 10**

*(Taiwan) Improving the Performing Rate of Enhanced Recovery After Surgery (Eras) in Patients Receiving Urological Surgery -A Preliminary Study*

**Chen Tien Mei, RN^1^, Lin Huei Wen, RN^1^, Guo Shu Liu, PhD^1^, Wang Yueh Mien, RN^1^**

^1^Taipei Medical University Hospital, Taipei, Taiwan

**Objectives:** Evidences represent that Enhanced Recovery after Surgery (ERAS) improved the rapid, uncomplicated recovery after surgery with benefits for patients while improving quality and saving costs. The results depend on an approach to teamwork and continuous audit, consisting of early ambulation, early removed of drains and tubes, early oral intake of fluids and solids, and appropriate pain management. The study aimed to use a multiple intervention program for improving the performance of ERAS protocol in patients with receiving urological surgeries.

**Methods:** In a cross-sectional survey, the low performing rate (64.1%) of ERAS protocol was determined in an urological surgery ward in a teaching hospital in northern Taiwan in March 2018. Through the cause and effect analysis and roots cause analysis in the lower performing rate of ERAS, the influence factors were that nurses had the deficient knowledge of ERAS, and they didn’t understand to use the complicated context of patient education about ERAS as well as the standard nursing care procedure of ERAS protocol. The intervention program included that nurses’ education program consisting of ERAS protocol about patients receiving urological surgery with general anesthesia as well as standard nursing procedure of ERAS, developed patients education of ERAS for urological surgery, and evaluating the standard operating ERAS of nursing care for those patients. All participants completed the satisfaction questionnaire of nursing care in hospital time.

**Results:** 480 patients conducted from March 2018 to February 2019. The performing rate of ERAS protocol significantly improved from 64.1% to 96.7% in patients receiving urological surgery with general anesthesia. In addition, the length of days in hospital reduced around 1 day from the mean of 3.65 days to 2.64 days in this study. They also reported an increasing score of the satisfaction questionnaire about nursing care in the hospital time from 86% to 95%. The mean scores of pre-post exams about nurses’ knowledge of ERAS were significant increased from the exam score of 80 to 94.8.

**Conclusion:** The results represented that this intervention was benefits of the increasing performance of ERAS protocol for patients with urological surgeries while improving healthcare quality and reducing the hospital time. Nurses could also increase knowledge about ERAS through the education program. It would further enhance the quality of patient care and saving healthcare money.

**Abstract 11**

Patient and caregiver experiences in home care with implantable Ventricular Assist Devices in Japan -Examination of illness beliefs using the lifeline method

**Kayo Nagano, PhD, MSN, RN1, Midori Kamizato, PhD,PHN, RN^1^**

^1^Okinawa Prefectural College of Nursing, Naha City, Japan

**Background:** In Japan, the number of transplant donors is very small, and patients needing heart transplantation are required to wear an implantable Ventricular Assist Device (VAD) and face a long waiting period of 3 to 5 years. However, few research reports have revealed patients’ long-term home care experiences in Japan, and it is difficult to say that there is currently enough support for caregivers. In long-term home care with VADs in Japan, it is necessary to deepen the understanding about the experiences of patients and caregivers and to examine their support.

**Purpose:** To clarify the experience of process for patients and caregivers in home care from worsening heart failure to VADs.

**Methods:**We targeted patients and caregivers who had been discharged from home and experienced home care for more than 3 months with VADs in a prefecture in Japan. The purpose of the research was explained, and those who provided consent were regarded as the research participants. We described the highs and lows from the time of worsening heart failure to the interview day as a single trajectory (lifeline method), and, based on that, we conducted separate semi-structured interview surveys with the patients and caregivers. The obtained results were analyzed according to the time of heart failure worsening, that of wearing the VAD, and that of home care, as well as by caregiver attributes.

**Results:** We interviewed 7 VAD patients and 7 primary caregivers. The patients’ trajectories dropped at the time of heart failure worsening and rose after VAD surgery, but dropped from the second year to the third year of home care and rose again after the third year. A patient in the third year after wearing VAD has experienced the strength of thinking “keeping up the fight and fighting the disease,” as well as a mental struggle after a painful period of keeping up the fight while awaiting heart transplantation. On the other hand, the caregivers’ trajectories showed various ups and downs, and the narrative of the experience was characterized by their patients’ attributes. In particular, the lifelines of the patient and the mother of caregiver drew similar trajectories and showed a disease experience that closely reflected the patient’s beliefs.

**Conclusions:** The long-term transplant waiting period must be supported by considering the patients’ experiences based on the disease and VAD timeline as well as the caregivers’ experiences based on their patients’ attributes.

**Abstract 12**

*Cultural Care for Dying Patients in Okinawa and Taiwan*

**Sayuri Jahana, PhD, RN^1^, Midori Kamizato, PhD,RN^1^**

^1^Okinawa Prefectural College of Nursing, Naha, Okinawa, Japan

**Background:** Understanding of cultural background of patients and families is crucial in providing care. What patients and families believes is heavily influenced by their culture especially when death is approaching.

**Objective:** The purpose was to explore how nurses should care for dying patients and families in Okinawa and Taiwan

**Methods:** Eight nurses in hospitals and palliative Care Units (PCUs) were interviewed about how they have given culture-based care for dying patients and bereavement family. Data analysis was conducted by using a qualitative descriptive method. The research was conducted after approval by the College ethics review panel.

**Results:** People of Okinawa Islands believed that death was considered as a rite of passage where the spirit and body separated from each other. When a person died outside of his home and the body was brought home for the funeral, it was believed that the spirit remained at the at the location of his death. To ensure that the spirit returned home, a ritual called “NUJIFA” was performed at the site of the death. When patient passed away, the nurses in the hospital and PCUs in Okinawa offered the family chance to perform NUJIFA by the family itself or the family who was taught by the funeral parlor. In addition, nurses in PCU allowed family to perform NUJIFA not only the patents room but also the bathroom which the family though necessary. 

In Taiwan, people wish to die at home because they believed a soul would not be able to return from a hospital. When the patient passed away in the hospital and PCU, nurses offered the family time and space to pray to Amida Buddha for 8 hours after patient’s death in the special room. In the ICU of the hospital, the nurse played a chant Amida Buddha for a dying patient who Buddhist. Many individuals of Buddhist faith may attribute pain and suffering to bad karma. A Buddhist patient in the PCU never used pain medication despite severe pain because of it. She just prayed and endured. A Nurse told a metaphor about God’s sending people for help, and she finally accepted pain medication.

**Conclusion:** Similar culture-related care was found in both Okinawa and Taiwan. It was similar for both them that they wanted to die at home and wanted the ritual for the souls. It is important to be sensitive to the culture and belief in order to provide care for dying patient and family coping with bereavement. Future studies are needed in order to develop a care program for nurses. It is important to be sensitive to the culture and belief in order to provide care for dying patient and family coping with bereavement.

**Abstract 13**

The Achievement and Challenges of Graduates of the Nurse Bridge Program in Cambodia

**Hyang-yon Rhee, PhD^1^, Suhyun Bae, Ed. M.^1^, Sook Ja Yang, PhD^1^, Sung-Heui Bae, PhD^1^, Bomi An, MSN^1^**

^1^Ewha Womans University, Seoul, S. Korea

This study aims to explore how graduates of the EWHA-Cambodia UHS (University of Health Science) Partnership Program perceive their experience and effect of the program. We performed the study as a part of the evaluation on the 2nd project (2017-2018) running a 2-year nurse bridge program for a bachelor’s degree at UHS for nurses with ADN. The purpose of the program was to strengthen nursing and research competency of the nursing leaders in Cambodia. 

The data of this study consisted of a survey and focus group interviews. The participants of the survey were 38 out of 45 NBP graduates from the 1st project (2015-2016) of the nurse bridge program. The questionnaire consisted of questions about the most memorable and challenging aspects of the program. The collected questionnaires were analyzed using SPSS. The participants of the Focus Group Interview (FGI) were three nurses who participated in the survey. FGI was conducted to explore participants’ perception of the program. Interview questions included the satisfaction with the program and change through the program in terms of nursing, research, and clinical competence. The interview was recorded and transcribed for detailed analysis. 

According to the results of the survey, 31.8% of the participants responded that the performance of the research, upgraded knowledge, and group activity were the most memorable. 51.8% of the participants answered that the most challenging in the program were language and research. Graduates who earned a bachelor’s degree through the program reported that they received additional benefits such as promotion, salary increases, and employment. They described that they could use what they learned in those lectures and minimize their mistakes in caring for patients after the program. The participants perceived that they had improved not only nursing but also English and IT skills. They suggested that lecturers of the program should have better English skills so they can communicate more with students. Furthermore, they wished that the master’s course in this program would be open so that they could pursue a higher degree in it. The results show that the nurse bridge program plays a significant role for the vulnerable nurses in improving their competences as nursing leaders in Cambodia. This study implicates that the nurse bridge program will improve the level of nursing and healthcare in Cambodia by creating nursing professionals with nursing and research competences.

Implications for Nursing is to improve the level of nursing and healthcare in Cambodia by creating nursing professionals with nursing and research competences.

**Abstract 14**

Type D Personality and Caring Ability in Nursing students : The Mediating Effect of Emotional Intelligence and Resilience 

**Juyeon Lee, MSN, RN^1^, Sookyoung Kim, PhD, RN^1^**

^1^CHA University, Pocheon, S. Korea

**Background:** Nursing is a practical discipline that cares for each individual with his or her own unique characteristics and circumstances in mind. In recent years, caring has attracted attention as a major concept in nursing. According to precedent research, nursing student’s caring ability was reported to be affected by emotional intelligence and resilience. However, there hasn’t yet been studies on whether the emotional intelligence and resilience have mediating effects on the relationship between nursing student’s type D personality and caring ability.

**Purpose:** Aim of this study was to examine mediating effect of emotional intelligence and 

**Methods:** The participants of this study were 3rd and 4th grade students in two nursing departments in G-Do, who were interested in understanding the purpose of the study and volunteering to participate in the study. Type D personality, caring ability, emotional intelligence and resilience were measured by using 278 questionnaires conducted from October 1st, 2018 to October 12th, 2018. Data were analyzed using Pearson’s correlation and multiple regression involving Ha* Process Macro.

**Results:** As a result of regression, in step 1-1, type D personality have a significant effect on emotional intelligence (B = −6.80, p < .001). In step 1-2, the type D personality have a significant effect on resilience (B = −6.77, p < .001). In step 2, type D personality and emotional intelligence have a significant effect on caring ability (B = −7.20, p = .001). Emotional intelligence has a significant mediating influence on the relationship of type D personality and caring ability while resilience doesn’t.

**Conclusion:** Through this study, in order to improve the caring ability of nursing students, it is necessary to identify the type D personality of nursing students and to apply the intervention program for the type D personality and to develop and apply the emotional intelligence enhancement program.

**Abstract 15**

Educational attainment predicts longitudinal change in insomnia severity in nurses 

**Hui-Ling Lai, PhD, MSN, RN^1^, Chiung-Yu Huang, PhD, MSN,BSN,RN^2^**

^1^Tzu Chi University, Hualien, Taiwan, Taiwan

^2^I-Shou University, Kaohsiung City, Taiwan

**Background:** Insomnia of nurses results in poor health and patients outcomes. However, research related to insomnia in newly graduated nurses in a longitudinal manner is limited worldwide. A most recent systematic review study have suggested that longitudinal studies were needed to identified what factors make individuals more vulnerable to sleep development.

**Objectives:** The objective of this study was to examine the course and factors associated with longitudinal changes in insomnia severity in newly graduated nurses. 

**Methods:** The sample was consisted of 200 participants generating 800 observations of severity of insomnia during their first year nursing career. The participants who were recruited from two hospitals in Taiwan completed a package of instruments measured at study entry and 3-monthly to one year. While accounting for the repeated measures within participants over the duration of first year after graduation from nursing schools, growth curve modeling (GCM) and growth mixture model (GMM) analysis were performed to assess the nature of insomnia patterns and the magnitude of growth curve. Descriptive analyses were conducted using SPSS, and Mplus 8 was used for GCM and GMM.

**Results:** Baseline insomnia severity scores were available for 279 nurses, of whom 65.6% had insomnia. The results of multiple regression GCM indicated that educational attainment significantly predicted the growth rates of insomnia severity in newly graduated nurses. There was a signi?cant difference in growth rates of insomnia severity between those who had BSN degree (bachelor’s of science in nursing) and those who had AND (associate’s degree in nursing) degree (p = .012) with those having AND degree being worse insomnia across time points. Moreover, occupational stressors at each time point were significantly associated with worse insomnia severity across times (all p < .001). 

**Conclusions:** The findings contributed to the knowledge about that the educational attainment predicted the growth rates of insomnia in nurses. Further studies should consider more specific social and organizational factors so as to gain insight into all of the complexities of severity of insomnia, and consider developing different intervention strategies for this homogeneous group of insomniac nurses. Using growth trajectories as predictors to examine the impacts on nurses’ retention is also needed to be studied.

**Implications for Nursing:** A higher level of educational attainment made nurses more resilient to the development of severity of insomnia. Attention to these aspects could potentially contribute to a better management of stress and insomnia symptoms in newly graduated nurses.

**Abstract 16**

Specialty of the care depending on stage aggravation of dementia - Points of attention of Stage Approach to Dementia on healthcare professionals 

**Miho Takami, PhD^1^, Yoshiko Nakasuji, MSN, CNS^1^, Akashi, Japankie Kayano, MSN^1^, Yuho Muto, BSN^1^**

^1^*College of Nursing Art & Science, University of Hyogo, Akashi, Japan*

**Background:** It is difficult to treat a symptom of dementia, there is a compelling need for making clear the care practice of match the stage and living conditions for people with dementia.

**Purpose:** The purpose of this study is to identify what healthcare professionals engaged in dementia care (e.g. nurses, care-workers) focus on when they interact with people with dementia, their families and physicians, in each (i.e. mild, moderate, or severe) stage of dementia.

**Methods:** This study was Qualitative Research, and data were collected at medical institutions, healthcare service facilities for the elderly, nursing homes, and group homes. For this study, 13 nurses and 9 healthcare workers were interviewed. The interviews were analyzed using content analysis.

**Results:** The results showed that in the period from the onset of dementia to mild stage, healthcare professionals engaged in dementia care focused on listening carefully to dementia patients and their families to understand their situations and watchful waiting until intervention is necessary. However, professionals seemed to pay little attention to interventions that could have a meaningful therapeutic effect on dementia. In the moderate stage, professionals focused on discovering the hidden “true personality” of dementia patients, by analyzing information on behaviors displayed, i.e. behavioral and psychological symptoms of dementia (BPSD) assessment. Regarding efforts to create a comfortable therapeutic environment for patients, professionals made adjustments to the care environment, focusing on the “human living,” to ensure that family and physician involvement in dementia care is not interrupted. In the moderate to severe stage of dementia, professionals focused their efforts on understanding dementia patients’ intentions and worked with physicians to help dementia patients experience a comfortable end of life, and their families to spend a peaceful time with them. However, the results were unclear concerning preventive care for complications and concrete support for decision-making about care.

**Conclusions:** These results show that professionals engaged in dementia care are required not only to possess the ability to act and management capability so that they can deliver necessary care for dementia patients and their families in cooperation with multi-professional team members, but also to comprehensively assess dementia patients so as not to deteriorate their quality of life.

**Abstract 17**

Action Research Aimed at Improvement of Mental Healthcare Services in Communities where Social Resources for Mental Healthcare and Welfare Services are Limited 

**Kaori Ishikawa, PhD^1^, Reiko Kuzuya, MSN^1^, Yoshimi Endo, PhD^2^**

^1^Gifu College of Nursing, Hashima, Gifu, Japan

^2^Osaka University, Suita, Japan

**Background:** In Japan, a policy was proposed to discharge psychiatric inpatients within 1 year and to create a community health care for those patients. However, it is difficult to provide support for them in communities with scarce social resources. 

**Purpose:** The purpose of this research is to identify and resolve issues for supporting and enabling people with mental illness to live fuller lives in communities with scarce social resources, and to improve mental healthcare services in such communities through cooperative programs between researchers and local nurses.

**Methods:** To understanding issues of support of people with mental illness, we conducted group discussions and interviews with specialists in the community (Action 1). We held three workshops. After the workshops, we conducted a questionnaire to evaluate the participants’ experiences and satisfaction (Action 2). To clarification of the roles of public health nurse to support people with mental illness, we conducted observations of her activities, and held interview with other professionals (Action 3). To clarification home-visit nursing care for people with mental illness, we analyzed three cases of home-visit nursing care and the data from interviews with home-visit nurses (Action 4).

**Results:** In action 1, the issues of mental health were mental healthcare for aged people and children, measures for untreated mental ill people and so on. Supporters could not engage in enough collaboration with other professionals. Public health nurse thought that their roles in supporting people with mental illness were unclear. Home-visit nurses did not feel confident in their care for mental ill. In action 2, participants shared the strengths of and issues of their mental healthcare services and were able to empower each other through the workshops. In Action 3, the six roles of public health nurses became clear, including network development of a basis for support and so on. In action 4, four effective care were defined, including user- and family-oriented care, collaboration with the user’s family and so on. After completing Actions, public health nurse and home-visit nurses, decided to hold support meetings on a regular basis.

**Conclusions:** These Actions led to collaboration in actual cases. We were able to suggest the importance of care services for people with mental illness to live in communities with limited social resources for mental health from the standpoint of public health nurse and home-visit nurse.

**Abstract 18**

Developing A Decision-Making Support System For Health Literacy In Multi-ethnic Females 

**Hsiu-Min Tsai, PhD, RN^1^, Po-Hsiang Huang, Senior Student^1^, Ya-Ni Wang, MSN, RN^1^, Yu-Wen Chou, MSN, RN^1^**

^1^Chang Gung University of Science and Technology, Guishan Dist., Taoyuan, Taiwan 

**Background:** Health literacy influences health behaviors, health utilization, patient-physician interaction, disease prevention and treatment. Women’s health literacy not only influences their personal health behaviors but also influences health of their family. 

**Purpose:** The purpose of this study is to evaluate the effectiveness of the females’ health literacy decision-making support system (DMSS) in multi-ethnicity females in Taiwan. 

**Method:** A pre-experimental method with pre-posttest in one group was conducted. Participants who are Ho-Lo, Hakka, Aborigines, Mainlanders, and Southeast Asian were recruited using convenient sampling. The questionnaires in the study contained demographic data sheet, General Self-Efficacy Scale, Self-Directed Learning Instrument, Adolescents’ Health-Promoting Behavior Scale and Taiwan Female Health Literacy Scale. The data analysis was conducted by descriptive statistics and paired t test. 

**Results:** A total of 1511 multi-ethnic females visited the studied DMSS, 99 completed both pretest and posttest. Large proportion of the participants was Taiwanese, single, employed, had an educational level equal or higher than college and university, had a family income between 50,000 and 100,000 NTD. In terms of learn intentions, the Ho-Lo has the highest intention to learn the modules of menstrual health, menopause health and reproductive health. The Hakka has the highest intention to learn the women’s specific disease module. New immigrants have the highest intention to learn the female cancer module. In terms of behavior patterns of learning, the Ho-Lo and Hakka tend to conduct tests first and then select learning modules. Aboriginal people and new immigrants tend to enter the learning theme before conducting the subject test. According to the comparative analysis of pre and posttest data, participants’ self-efficacy, self-learning, health promotion and female health literacy were significantly different (p < .01). 

**Conclusion:** These results indicated that the designed DMSS was effective in increasing females’ health literacy and health promoting behaviors. The females’ health literacy DMSS might be widely used by all females. For further implementation, healthcare providers need recognize that there are different learning intentions and behavior patterns of health literacy among multi-ethnic groups of women in Taiwan. This study was funded by the Ministry of Science and Technology (106-2511-S-255-001,106-2511-S-255-003-MY3).

**Implications for Nursing:** The designed DMSS was effective in increasing females’ health literacy and health promoting behaviors and might be widely used by all females. For further implementation, healthcare providers need recognize that there are different learning intentions and behavior patterns of health literacy among multi-ethnic groups of women in Taiwan.

**Abstract 19**

Development and Application of a Cognitive Behavior Therapy Program for Patients with Fibromyalgia Syndrome 

**Kyoung ran Kong, PhD, RN^1^, Eun Nam Lee, PhD, RN^2^, Sung Gum Kang, PhD, RN^3^**

^1^Ulsan College, Ulsan, Ulsan, S. Korea 

^2^Dong A University, Pusan, S. Korea

^3^Gim Hae College, Gim Hae, S. Korea

**Objectives:** This study developed a cognitive behavior program aimed at altering the physical conditions, emotions, and behaviors of patients with fibromyalgia syndrome, and confirmed the program’s clinical applicability via hypothesis testing. 

**Methods:**The cognitive behavior program was developed by analyzing previous studies, conducting in-depth interviews with fibromyalgia syndrome patients, drawing on cognitive behavior theory to establish the program contents, recruiting experts to test its validity, and conducting a preliminary survey. In order to confirm the effect of the program, this study used a randomized controlled trial design. The subjects were outpatients who had been diagnosed with fibromyalgia syndrome in University Hospital D, Busan. The 34 patients in the experimental group took part in the cognitive behavior program, which comprised 8 sessions (90 to 120 minutes) based on cognitive behavior theory, delivered over 8 weeks. The 34 patients in the control group were allowed to participate in the same program after the experimental group’s intervention had ended. The collected data were tested for the Kolmogorov–Smirnov test via SPSS Statistics 24.0. Hypothesis testing was performed using the repeated measures ANOVA and the Friedman test. 

**Results:** The analysis revealed significant differences between the experimental group (who participated in the cognitive behavior program) and the control group (who did not participate) in positive automatic thoughts (F = 14.24, p < .001), negative automatic thoughts (F = 3.46, p = .036), pain (χ^2^ = 18.38, p < .001), fatigue (F = 9.34, p = .000), depression (χ^2^ = 16.99, p < .001) and interpersonal relationships (χ^2^ = 23.18, p < .001). However, there was no significant difference between the groups in terms of sleep disorders (F = 1.15, p = .289). 

**Conclusions:** In conclusion, the changes in thought caused by the cognitive behavior program for patients with fibromyalgia syndrome appears to have had a positive effect on their physical conditions, emotions, and behaviors, which is in line with the cognitive behavioral model. It is expected that this program can be used in the future as an effective nursing intervention program to help fibrmyalgia syndrome patients improve their disease conditions.

**Abstract 20**

A meta-analysis for exercise effects in ankylosing spondylitis: Focused on the pulmonary function 

**Eun Nam Lee, PhD^1^, EunJeong Kim, Doctoral student^1^, Eun-Jeong Yu, Doctoral student^1^**

^1^Department of Nursing, Dong-A University, Seo-gu, S. Korea

**Purpose:** The ankylosing spondylitis (AS) is characterized by stiffness and decreased mobility of the spine due to inflammation and structural damage of the spine. If the movement of the chest wall is restricted, it will have a negative effect on the functional part of the lung. These functional impairments and disability are serious problems that lead to decrease of quality of life. Although exercise has been recommended as a nonpharmacologic treatment to manage it, attempts to understand comprehensively the effects of exercise interventions that associated with restricted pulmonary functions were lacking. The purpose of this study is to analyze the effects of exercise on pulmonary function in AS. 

**Methods:**We searched MEDLINE, EMBASE, CENTRAL, CINAHL (through Jan 2019) to identify RCTs on the effectiveness of exercise therapy on pulmonary function adults with AS. Three reviewers independently extracted data and assessed methodological and therapeutic validity (using the risk of bias scale in Cochrane).

**Results:** The search retrieved 353 articles of which studies - describing 13 interventions (438 patients) – fulfilled our inclusion criteria. VO2 peak values were significantly higher in studies involving aerobic exercise (3.446 (0.24; 0.88)), FVC were found to be effective in the inspiratory muscle training group (3.459 (0.47; 1.69)). CE had a significant effect on the most exercise interventions (6.520 (0.41; 0.75)). As a secondary variable, BASFI was effective in all studies (−6.597 (−0.67; -0.37)) 

**Conclusion:** As a result of the study, all studies aimed to improve the functional status of people with AS, but there was no study focusing on pulmonary function, so it was difficult to grasp the close relationship between the variables. Even though these studies were RCTs, exercise modality like monitoring, adequate dosing, and personalization were rarely addressed in the programs. The exercise form was mainly aerobic exercise and strength exercise, and several types of exercise were combined. The pulmonary involvement is common in AS and might have disturbed functionality and exercise modality. This study was meaningful in that it provided the idea of how to view pulmonary function as a major variable to evaluate the effect of exercise and to determine the most useful type of exercise program for AS patients. 

**Abstract 21**

Structural Equation Modeling of Health-Related Quality of Life in Patients with Systemic Lupus Erythematosus: Application of Resourcefulness Theory 

**Eun Nam Lee, PhD, RN^1^, Eun Hui Choi, PhD, RN^2^, Moon Ja Kim, MSN, RN^1^**

^1^Dong-A University, Busan, Busan, S. Korea

^2^Masan University, Changwon-si, S. Korea

**Objectives:** The present study is a structural equation modeling study that developed a hypothetical model to explain the health-related quality of life in lupus patients by using Zauszniewski’s resourcefulness theory and verified the goodness of fit (GOF) of the model.

**Methods:** According to the resourcefulness theory, the disease activity and social network of lupus patients were set as antecedent factors, positive cognition as a process regulator, and physical and psychological quality of life as indicators of quality of life to construct a hypothetical model in this study. Data collection was conducted with patients who were diagnosed with lupus and were receiving outpatient treatment at D University Medical Center located in B Metropolitan City in Korea from June 1 to August 30, 2018, and a total of 252 patients’data were included in the final analysis. The collected data were analyzed using SPSS statistics 24.0 and AMOS 25.0 program.

**Results:** Results of validity test of the hypothetical model revealed that the GOF was indicated by X2/df (2.51), SRMR(.06), RMSEA(.08), GFI(.92), AGFI(.87), TLI(.91), and CFI(.94), demonstrating that the figures met the recommended level. The results of the model analysis showed that the resourcefulness of lupus patients directly affected their physical and psychological quality of life, and that the disease activity degree did not affect their resourcefulness, but their social network did. Positive cognition was found to have a mediating effect on disease activity degree, social network, and resourcefulness. It was found that although the disease activity degree directly affected the physical and psychological quality of life, social network had a direct effect only on the psychological quality of life.

**Conclusions:** In conclusion, the resourcefulness of lupus patients affected their physical and psychological quality of life, and resourcefulness was influenced by disease activity degree and social network with positive cognition serving as a mediating factor in the process. Based on these findings, the authors of this study propose the development of research of interventions that can enhance the resourcefulness to improve health-related quality of life of lupus patients.

**Abstract 22**

A Novel Approach for Measuring Socioeconomic Factors Associated with Cardiovascular Health Among Older Adults in South Korea 

**Chiyoung Lee, MSN^1^, Eun-Ok Im, PhD, MPH, RN, CNS, FAAN^1^**

^1^Duke University, School of Nursing, DURHAM, USA

**Background:** Cluster analyses meaningfully interpret the interrelated socioeconomic status (SES) components of important health outcomes, enabling researchers to identify vulnerable groups. However, this approach has thus far been insufficient in the cardiovascular field. Older adults in South Korea comprise a unique group, with great socioeconomic variability.

**Purpose:** The study aimed to identify socioeconomic clusters of older adults and to compare cardiovascular outcomes among the identified clusters.

**Methods:** A cross-sectional analysis was performed using the data from 3303 older adults (over 65 years; 56.5% female) who participated in the Korean National Health and Nutrition Examination Survey (2016–2017). A two-step cluster analysis was used to identify older adults’ socioeconomic clusters based on eleven SES factors. Socioeconomic levels were assigned to the clusters contingent upon which SES variables were more prevalent in each cluster. A comparison test (Chi-squared and ANOVA test) was performed to validate the cluster solution and explore the differences between the identified clusters. In addition, logistic and linear regression analyses estimated the risk values for the prevalence of cardiovascular diseases (CVDs) and associated risk factors.

**Results:** A three-cluster solution was selected (p < 0.00): low (N = 715), middle (N = 1,425), and high socioeconomic (SES) groups (N = 1,163). After controlling for initial health status and health behavior, decreased odds of having diabetes (OR = 0.74, p < 0.01) and hypertension (OR = 0.70, p < 0.00) were found in the high SES group when compared with the middle SES group, and elevated odds of having hypertension (OR = 1.22, p < 0.00), being overweight (OR = 1.20, p < 0.05), and obese (OR = 1.43, p < 0.00) were found in the low SES group in the same comparison. Using a linear regression model, elevated risk differences in 10-year CVD risk levels (RD = 1.38, p < 0.01), total cholesterol (RD = 5.32, p < 0.01), and systolic blood pressure (RD = 2.88, p < 0.01) were observed in the low SES group, compared to the middle SES group. However, no significant differences were found in the prevalence of CVDs among clusters.

**Conclusion:** Understanding the potential combinations of SES risk factors could facilitate etiologic understanding of cardiovascular health. Furthermore, older adults of low SES groups should be a crucial target group for prevention and management of CVD in health promotion interventions.

**Implications for Nursing:** This study supported the feasibility of applying clustering to assess varying SES conditions that exist within an older adult population, a method which should be considered in future research planning.

**Abstract 23**

Future Directions in Nursing Research Across the Globe

**Eun-Ok Im, PhD, MPH, RN, CNS, FAAN**

Duke University, USA

**Abstract**: Researchers have emphasized the necessity of nursing research in the practice of professional nursing. Indeed, nursing research has provided solid grounds for evidence -based care that enhances health outcomes of individuals, families, communities and health care systems. Also, nursing research has shaped policies related to health care, within an organization, and at the local, state and federal levels. With advances in communication and transportation technologies, nursing has experienced globalization as in other fields. However, little has been discussed about future directions in nursing research across the globe. In general, the 2016 NINR strategic plan clearly prescribes future directions of nursing research, which include four focused areas of research including symptom science, wellness, self management, and end-of-life and palliative care. Characteristics and challenges of top NIH funded schools of nursing also provide directions for future nursing research. Also, current trends in nursing in general provide future directions for nursing research. The current trends in nursing include: (a) changing demographics; (b) increasing diversities and globalization; (c) technology innovation; (d) individualized/personalized care; (e) population health; (f) cost-effectiveness; (g) changes in health policies/regulations; (h) interdisciplinary collaboration; and (i) nursing workforce changes. Based on the discussion on the current trends, several recommendations for future nursing research across the globe are proposed: (a) promoting personalized health strategies; (b) promoting health and preventing illness; (c) improving quality of life for individuals with chronic conditions; and (d) end-of life and palliative care. 

**Symposium 1: Abstract 1 - Overview**

The HEARTS of Older Persons in Pittsburgh, PA and the Philippines: A Global Collaboration

**Rose Constantino, PhD, MSN, BSN, RN, JD, FAAN**

University of Pittsburgh School of Nursing, Pittsburgh, PA, United States

**Background:** Of the 48 million older adults (60 years and older) in the United States, an estimated 90% of them have one common desire: to age well, safely, in their own homes; their desire is also the most cost-effective for the states, and the nation. The National Academy of Medicine drew attention to the incapacity and inadequacy of health care systems to deliver high-quality services to the growing older population. We are presenting 2 Symposia from 2 research sites Pittsburgh, PA and Manila, Philippines as a global collaboration.

**Purpose:** Our purpose was to assess the HEARTS 

**Methods:** Research Design. A mixed method sequential transformative research design was used in this research. We used a community-based engaged research approach as older persons are often stereotyped, marginalized, and silenced in their homes, groups, and communities. 

**Data Analysis:** Quantitative. A detailed descriptive data analysis such as means, standard deviations, percentiles, ranges, and graphic presentations will be presented. Qualitative. The interview schedule generated robust information from the participants. For the Manila HEARTS, all conversations between investigator and participant were video-taped after informed consent.

**Results:** will be provided as each abstract is presented.

**Summary and** **Conclusions:** Symposium #1 has 4 abstracts 1) Community Engagement: A Crucial First Step in Community-Based Participatory Research, 2) The Association Between Experience of Abuse Sleep Quality among Older Adults in the Pittsburgh Area, 3) Harnessing the Internet, Mobile, and Wearable Devices to Facilitate Health Outcomes, and 4) The Association Between Experience of Abuse and Anxiety of Older Persons in the Pittsburgh Area. Symposium #2 has 4 abstracts 1) The Experience of Abuse and Resilience of Older Persons in Urban Philippines, 2) The Health and Experience of Abuse of Older Persons in the Philippines, 3) The Treatment of Social Networks and Safety Status of Filipino Older Persons, and 4) Identifying Interventions that Enhance Resilience in Filipino Older Persons. The population aged 60 and over is growing faster than all younger age groups globally. Aging is poised to become the most significant social transformations of the 21st century with major implications. Older persons are contributors to world development; their talents and abilities should be woven into policies and programs at all levels. Adding health to years is a global theme for 2020 and beyond.

**Symposium 1, Abstract 2**

Community Engagement: A Crucial First Step in Community-Based Participatory Research 

**Claudia Kregg-Byers^1^; Paul Scott PhD^1^; Dianxu Ren, PhD, MD^1^; Rose Constantino, PhD, JD, MSN, BSN, RN, FAAN^1^**

^1^University of Pittsburgh School of Nursing, Pittsburgh, PA, Pittsburgh, USA 

**Background:** The impact of elder abuse could undermine self-confidence, emotional stability, self-esteem, and adaptability. While scholars have made significant progress in addressing elder abuse across different cultures, there is a paucity of study that uses the A for Accommodation-B for Building Bridges-C for Collaboration-D for Diversification in recruiting study participants as co-researchers. We used community-based participatory research critical to the development of innovative and effective community-based violence prevention strategies. 

**Purpose:** The purpose of this paper is to define, clarify, and magnify, community-based participatory engagement as a crucial first step in community-based research using the theoretical framework of Community-Based Engagement prior to our assessment of the HEARTS (Health, Experience of Abuse, Resilience, Technology use, and Safety) of older persons. 

**Methods:** This study uses a mixed methods design. We visited and attended community activities in 6 Pittsburgh areas: South Hills, North Hills, East Liberty, Squirrel Hill, Homewood, and Lincoln-Larimer. From the community gatherings, older persons aged 60 years and older were recruited in the Pittsburgh, PA area after receiving IRB approval. 

**Results:** As members of the Community Engagement Panel (CEP), one of the researchers in her talk invited those present at the first community meeting to join hands as members of the CEP and that they are not only participants in research but co-researchers. The group developed a 4-member research CEP. This 4 member CEP led the entire group of participants in defining, clarifying, magnifying, and engaging participants to ask questions regarding the research problem and answer pointed questions such as, how long are we going to do this research, what good can we get from it? These were answered by the CEP and the PI or Co-I. 

**Conclusions:** The topics discussed were sensitive, the hesitancy of people of color to be involved in research of any type, made it difficult to ascertain clear suggestions from participants regarding interventions that would enhance self-sustained engagement. Engaging neighbors and communities in research as partners, as opposed to the limited role of research participant, create more relevant outcomes to help resolve the science: community gap. 

**Symposium 1, Abstract 3**

The Association Between Experience of Abuse and Sleep Quality among Older Adults in the Pittsburgh Area

**Vivian C. Hui, BSN, RN^1^; Paul Scott, PhD^1^; Dianxu Ren, PhD, MD^1^; Rose Constantino, PhD, JD, MSN, BSN, RN, FAAN^1^**

^1^University of Pittsburgh School of Nursing, Pittsburgh, United States

**Background:** Elder abuse is a global public health issue that has reached alarming levels with high prevalence rate globally. The impact of elder abuse could undermine self-confidence, emotional stability, self-esteem, and adaptability. While scholars have made significant progress in addressing elder abuse across different cultures, there is a paucity of study examining the relationship between the experience of abuse and sleep quality. In older persons, sleep is a diminishing commodity. The consequences of sleep disturbances and sleep-related impairments can be health- and life-threatening. 

**Purpose:** The purpose of this study is to explore the association between experience of abuse and sleep disturbance among older persons in the Pittsburgh area.

**Methods:** We recruited 34 older persons aged 60 years and older in Pittsburgh. Two questionnaires EASI (Elder Abuse Suspicion Index) and PROMIS (Patient-Reported Outcomes Measurement Information System) were distributed to evaluate the experience of abuse and general health function like anxiety, depression, fatigue, sleep disturbance, sleep-related impairment, and physical function. Quantitative data were analyzed using a detailed descriptive and inferential statistical analysis. 

**Results:** Among the PROMIS variables, sleep disturbance was the highest rated health functional problems with mean = 10.19 and SD = 3.297. Results indicate that there are significant negative correlations between sleep quality and emotional abuse, r <  -0.301, p = 0.044, sleep quality and financial abuse, r = −0.298, p = 0.046, as well as between sleep quality and physical abuse, r = −0.366, p = 0.018. Additionally, we find significant negative correlations between the extent to which individuals report feeling refreshed after sleeping and the experience of emotional abuse, r = −0.345, p = 0.023; financial abuse, r = −0.308, p = 0.038; and, physical abuse, r = −0.376, p =  0.014. Participants who experienced abuse were prone to have lower quality and less refreshing sleep. 

**Conclusions:** This research elicited data that older persons with abused experience in the Pittsburgh area are mostly affected by sleep disturbance. Given these findings, we strongly suggest that future research could harness technology such as a wearable device to explore intervention strategies to prevent health consequences of sleep disturbance and sleep-related impairments in older persons experiencing abuse.

**Symposium 1, Abstract 4**

Harnessing the Internet, Mobile, and Wearable Devices to Facilitate Health Outcomes

**Linden Wu, BSN, RN^1^; Paul Scott, PhD^1^; Dianxu Ren, PhD, MD^1^; Rose Constantino, PhD, JD, MSN, BSN, RN, FAAN^1^**

^1^University of Pittsburgh School of Nursing, Pittsburgh, United States, 

**Background:** The impact of trauma and context on survivors’ ability to communicate and seek help from healthcare providers is immeasurable. The Internet, mobile, and wearable devices and accessories are rampantly used as conduits to prevent and manage chronic illnesses, violence, and trauma and facilitate dialogue between health providers and traumatized populations globally. Given the global challenge of an ever-expanding demand for healthcare management, our research team explored how to harness Email and text messaging, and WATCH (Wearable Accessory To Call for Help) to deliver HELP (Health, Education on safety, Legal rights, and Privileges) to women in Intimate Partner Violence (IPV). 

**Purpose:** Our purpose was to 1) explore email, text messaging, and WATCH as a method of delivering HELP, and 2) assess which method is efficient and effective. We used Disruptive Innovation (DI) as our conceptual framework (Christensen, 2012). 

**Methods:** We used mixed methods design in data collection and data analysis. Quantitative data were collected using self-report questionnaires and qualitative data were collected via email and text messaging interviews. Quantitative data were analyzed using a detailed descriptive analysis and comparisons of the effect of the intervention used the intention-to-treat principles. Qualitative data were analyzed using a phenomenological approach.

**Results:** The HELP intervention when delivered via email (1) decreased anxiety (diff.3.6%), depression (diff. 3.8%), and anger (diff. 4.3%), and (2) increased social support (diff. 14.5%). Qualitatively, the HELP information and intervention was shown to be feasible, acceptable, and effective among IPV survivors in the email participants. Results for the text messaging intervention showed that there was an increase in knowledge level pre to post-test scores, from 2.00 ±  1.00 to 2.7 ±  0.48 (p < 0.001) and confidence level pre to post-test score increased from 2.89 ±  0.60 to 3.30 ±  0.68 (p < 0.001). Only the results from the email and text messaging projects are reported here.

**Conclusions:** Barriers to email are that each user must have their own Internet connectivity and must be proficient in using the computer. These barriers are resolved in mobile and wearable devices. We plan to develop the WATCH4HELP (Wearable Accessory To Call for Help). prototyping of the WATCH4HELP is in the process. 

**Symposium 1, Abstract 5**

The Association Between Experience of Abuse and Anxiety of Older Persons in the Pittsburgh Area

**Jordan Cobb, Student^1^; Paul Scott, PhD^1^; Dianxu Ren, PhD^1^; Rose Constantino, PhD, JD, MSN, BSN, RN, FAAN^1^**

^1^University of Pittsburgh School of Nursing, Pittsburgh, United States

**Background:** Elder abuse is a multidimensional phenomenon that encompasses a broad range of behaviors, events, and circumstances. A substantial research has been working on the risk factors, prevalence, and consequences. There is a paucity of study accessing the relationship between the experience of abuse and the mental health of older persons. The prevalence of anxiety disorders in older people range from 1.2% to 15% in the community, and up to 28% in clinical settings.1,2 Older adults experience more stressors, in particular, loss of an intimate partner, illness, disability, fears of being a burden on others, impending mortality and reduced financial support. The consequences of anxiety and related health issues are far-reaching and life-threatening. Nevertheless, evidence-based research regarding the anxiety level in older adults is not significant in our review of the literature. 

**Purpose:** The purpose of this study is to explore the association between experience of abuse and anxiety level among older persons in the Pittsburgh area.

**Methods:** Two questionnaires EASI (Elder Abuse Suspicion Index) and PROMIS (Patient-Reported Outcomes Measurement Information System) were distributed to evaluate the experience of abuse and general health function like anxiety, depression, fatigue, sleep disturbance, sleep-related impairment, and physical function. Quantitative data were analyzed using a detailed descriptive and inferential statistical analysis. 

**Results:** Findings indicate that there are significant negative correlations between fearful level and physical abuse, r <  -0.280, p = 0.054, fearful level and financial abuse, r = −0.406, p = 0.009. Additionally, we find significant negative correlations between the extent to which individuals report hard to concentrate on anything except their anxiety and the experience of physical abuse, r = −0.318, p = 0.035; and, financial abuse, r = −0.324, p = 0.033. participants who experienced abuse were prone to suffer from anxiety more. 

**Conclusions:** The paucity of elder abuse and anxiety level shed light on the urgency to evaluate current intervention direction on violence against the elderly. This research elicited data that older persons with abused experience in the Pittsburgh area are anxious than those without abuse experience. Future research can explore the emotional need and support towards older abused adults with holistic care and human interaction.

**Symposium 2: Abstract 1 - Overview**

The Effects of Technology-Based Interventions on Health Outcomes

**Wonshik Chee, PhD**

Duke University, Durham, NC, USA

**Overview:** With advances in computer and mobile technologies, the use of technology-based interventions has drastically increased in recent years. Compared with conventional interventions, technology-based interventions are reported to be effective in providing information and coaching/support mainly due to easy access without time or cost constraints. However, little is still known about the effectiveness of technology-based interventions on health outcomes. 

**Purpose:** This symposium aims to provide an open forum to discuss the effects of technology-based interventions on health outcomes through three presentations from the same study that tested a technology-based coaching/support program for Asian American breast cancer survivors. 

**Methods and Results:** The first presentation shows the findings on the effect of sub-ethnicity on pain and symptom experience (pain management, symptom management, pain experience, and symptom experience) of Asian American Breast Cancer Survivors and to determine the multiple factors influencing the relationships. This presentation is to show the pre-test findings on the participants’ health outcomes in the study. The second presentation is about the effect of the technology-based coaching/support program on menopausal symptoms of Asian American breast cancer survivors. The intervention group showed a significant decrease in the distress scores of menopausal symptoms over time: physical (β = −0.07, p = 0.08), psychological (β = −0.13, p = 0.05), psychosomatic (β = −0.17, p = 0.06), and total symptoms (β = −0.19, p = 0.01). The final presentation provides the findings on the efficacy of a technology-based intervention on improving cancer pain and its accompanying symptoms of Asian American breast cancer survivors. Only the intervention group showed a significant decrease in the total symptom severity scores between pre-test and post-3-months (? = −0.30, p = .0125). Through this symposium, implications for future technology-based interventions for breast cancer survivorship are proposed.

**Symposium 2: Abstract 2**

Subethnic Differences in Pain and Symptom Experience among Asian American Breast Cancer Survivors 

**Chi-Young Lee1; Sangmi Kim PhD, MPH, RN^1^; Wonshik Chee, PhD^1^; Eun-Ok Im, PhD, MPH, RN, CNS, FAAN^1^**

Duke University, Durham, NC, Durham, USA

**Purpose:** This study aimed to examine the effect of sub-ethnicity on pain and symptom experience (pain management, symptom management, pain experience, and symptom experience) of Asian American Breast Cancer Survivors (AABCS) and to determine the multiple factors influencing the relationships.

**Methods:** This was a secondary analysis of the data from a larger study on the survivorship experience of AABCS; only the data from 94 women were used. Multiple questions on background characteristics, the Perceived Isolation Scale (PIS), the Personal Resource Questionnaire (PRQ-2000), the Memorial Symptom Assessment Scale-Short Form (MSAS-SF) were used to collect the data. The data were analyzed using chi-square tests, ANOVA, and hierarchical logistic and multiple regression analyses.

**Results:** Over 93% of the Japanese women were managing their pain while only less than 50% of the Chinese and Korean women were doing so (p < .01). They also experienced less pain (p = .03) and symptom distress (p = .02) than Chinese and Korean women. Being Japanese was a significant factor that influenced the women’s pain management (OR = 14.63 p = .02), symptom management (OR = 11.17, p = .02), pain experience (β = −0.32, p = .01), and symptom distress (β = −0.24, p = .01). Residential area significantly contributed to variances in symptom management (OR = 10.08, p < .01). In addition, religion and level of acculturation were significantly associated with symptom distress (β = −0.10, p < .01). 

**Conclusion:** The current study supported the influence of sub-ethnicity on AABCSs’ pain and symptom experience, with findings demonstrating that being Japanese was associated with better outcomes. Therefore, healthcare providers need to be aware of these sub-ethnic differences and tailor their cancer care while taking into account each sub-ethnic groups’ unique characteristics. Moreover, management or interventions for better survival outcomes in AABCS can be influenced by residential area, religion and the level of acculturation. Thus, these areas of influence should be considered in developing such interventions for AABCS. 

**Symposium 2: Abstract 3**

Technology-Based Information and Coaching/Support Program: The Effects on Menopausal Symptoms of Asian American Breast Cancer Survivors

**You Lee Yang, PhD, RN^1^; Eun-Ok Im, PhD, MPH, FAAN, CNS, RN^1^; Sangmi Kim, PhD, MPH, RN^1^; Wonshik Chee, PhD^1^**

^1^Duke University, Durham, NC, Durham, USA

**Objectives:** This study was to evaluate the effects of a technology-based information and coaching/support program on menopausal symptoms among Asian American breast cancer survivors. 

**Methods:** A pretest-posttest randomized controlled trial design was used. Ninety-one Asian American breast cancer survivors were included (intervention group 42, control group 49). The program was a theory-driven and culturally tailored technology-based program to enhance the survivorship of Asian American breast cancer survivors. Background characteristics, menopausal symptoms, and theory-based variables (attitudes, social influence, self-efficacy, and perceived barriers) were measured at the three time-points (pre-test, post-1-month, and post-3-months). For data analyses, an intent-to-treat mixed-model growth curve analysis was conducted using the SAS PROC MIXED.

**Results:** In a homogeneity test, there were no statistically significant differences in characteristics between the control group and the intervention group. For intervention group, there were significant decreases in the total distress scores of menopausal symptoms (β = −0.19, p = 0.01) and the sub-distress scores of menopausal symptoms: physical (β = −0.07, p = 0.08), psychological (β = −0.13, p = 0.05), and psychosomatic (β = −0.17, p = 0.06). The attitudes, social influences, and self-efficacy on breast cancer survivorship partially mediated the intervention effects on the distress scores of menopausal symptoms (p < .10).

**Conclusions:** The study results proved the effects of the technology-based information and coaching/support program on relieving menopausal symptoms of Asian American breast cancer survivors. Further research is needed to verify the effects of the program on diverse groups.

**Symposium 2: Abstract 4**

The Effects of a Technology-Based Program on Pain and Symptoms

**Wonshik Chee, PhD^1^; Sangmi Kim, PhD, MPH, RN^1^; You Lee Yang, PhD, RN^1^; Chiyoung Lee, MSN, RN^1^**

^1^Duke University, Durham, NC, Durham, USA

**Objective:** Pain and its accompanying symptoms are common problems, especially in the first few years of breast cancer survivorship after treatment as well as during the diagnosis and treatment process. Asian American breast cancer survivors reportedly have inadequate cancer pain and symptom management, subsequently reporting lower quality of life compared to other racial/ethnic groups. Technology-based programs could improve cancer pain and symptom management process. The purpose of this study was to examine the effects of a technology-based information and coaching/support program on cancer pain and its accompanying symptoms of Asian American breast cancer survivors. 

**Methods:**A randomized pretest/posttest group design was used for the study. The study included 42 Asian American breast cancer survivors in an intervention group and 49 in a control group. The technology-based program targeted to decrease cancer pain and its accompanying symptoms through providing information and coaching/support using computers and mobile devices. Background characteristics and menopausal symptoms were measured using multiple instruments at three time points (pre-test, post 1-month, and post 3-months). The data were analyzed using an intent-to-treat linear mixed-model growth curve analysis. 

**Results:** Only the intervention group showed a significant decrease in the total symptom severity scores between pre-test and post-3-months (? = −0.30, p = .0125). Although both groups tended to experience a decrease in the severity scores of physical symptoms over time (p = 0.0712), the decreasing rate was marginally greater among the intervention than the control group (p = 0.1032). However, cancer pain and psychological symptoms did not show significant group, time, and their interactive effects.

**Conclusions:** The findings supported that the program helped reduce the symptom distress of Asian American breast cancer survivors. Further studies with a larger number of Asian American breast cancer survivors are needed to confirm the findings.

**Symposium 3: Abstract 1 - Overview**

Assessing the HEARTS of Older Persons in Mandaluyong, Philippines

**Rose Constantino, PhD, MSN, BSN, RN, FAAN, FACFE**

University of Pittsburgh School of Nursing, Pittsburgh, PA, USA

**Background:** Of the 48 million older adults (60 years and older) in the United States, an estimated 90% of them have one common desire: to age well, safely, in their own homes; their desire is also the most cost-effective for the states, and the nation. The National Academy of Medicine drew attention to the incapacity and inadequacy of health care systems to deliver high-quality services to the growing older population. We are presenting 2 Symposia from 2 research sites Pittsburgh, PA and, the Philippines as a global collaboration.

**Purpose:** Our purpose was to assess the HEARTS (Health, Experience of Abuse, Resilience, Technology use, and Safety) of older persons. 

**Methods:** Research Design. A mixed method sequential transformative research design was used in this research. We used a community-based engaged research approach as older persons are often stereotyped, marginalized, and silenced in their homes, groups, and communities. Data Analysis

Quantitative. A detailed descriptive data analysis such as means, standard deviations, percentiles, ranges, and graphic presentations will be presented. Qualitative. The interview schedule generated robust information from the participants. For the Manila HEARTS, all conversations between investigator and participant were video-taped after informed consent.

**Results:** Will be provided as each abstract is presented.

**Summary and Conclusions:** Symposium #3 has 4 abstracts 1) The Experience of Abuse and Resilience of Older Persons in Urban Philippines, 2) The Health and Experience of Abuse of Older Persons in the Philippines, 3) The Treatment of Social Networks and Safety Status of Filipino Older Persons, and 4) Identifying Interventions that Enhance Resilience in Filipino Older Persons. The population aged 60 and over is growing faster than all younger age groups globally. Aging is poised to become the most significant social transformations of the 21st century with major implications. Older persons are contributors to world development; their talents and abilities should be woven into policies and programs at all levels. Adding health to years is a global theme for 2020 and beyond. 

**Symposium 3: Abstract 2**

The Experience of Abuse and Resilience of Older Persons in Urban Philippines 

**Tita Barcelo1; Dorothea Carino-Dela Cruz PhD^2^; Pearl Cuevas, PhD, RN^2^; Elvira L. Urgel, PhD, MAN, BSN, RN^2^**

^1^University of Pittsburgh School of Nursing, Pittsburgh, PA, USA

^2^Central Escolar University, Manila, Philippines

**Background:** With the rapid increase of older persons and the economic downturn in the Philippines, several Filipino households face the burden of supporting their older parents/grandparents and for those who are unable to find the means to support them, the older person is left neglected, financially exploited or abused. Elder abuse, according to the WHO, is a violation of human rights and a significant cause of illness, injury, loss of productivity, isolation, and despair among older persons globally. 

**Purpose:** The purpose of this research was to assess the HEARTS of older persons in the Philippines. An Acronym used for H-Health, EA- Experience of Abuse, R-Resilience, T-Treatment, and S-Safety. We aimed to identify interventions that will enhance resilience and address the experience of abuse among Filipino older persons in an urban city in the Philippines. 

**Methods:** We assessed the HEARTS of older persons by using PROMIS to measure health, the EASI to measure the experience of abuse, the CD-RISC to measure resilience and a socio-demographic questionnaire to assess the socio-demographic characteristics of participants. All measures were translated and back-translated into Tagalog, the Philippine language. All measures in English were found to have good psychometric properties. 

**Results:** The scores of abuse and resiliency correlation matrix show a correlation coefficient of 0.008 between the abuse score and resiliency score. The p-value score is 0.941 and is not significant at the set 0.05 level for the 2-tailed test. Therefore, results show no correlation between abuse and resiliency that means even though older persons have the possibility of abuse; this has nothing to do with their resiliency. 

**Discussion:** According to the World Report on Violence and Health, older people are isolated because of physical infirmities. Furthermore, loss of friends and family members reduces the opportunities for social interaction. Social isolation could be a harbinger of neglect. As people grow older, they become less resilient because of their frailty and inability to fend for themselves, thus they may receive a lower resilience score. 

**Conclusions:** The study gave evidence to the resilience of Filipino older persons even in times of verbal abuse. Thus, we conclude that primary prevention could be timely and effective in preventing the escalation of verbal/psychological abuse to physical, economic, or sexual.

**Symposium 3: Abstract 3**

The PROMIS and Experience of Abuse of Older Persons in the Philippines

**Dorothea Carino-Dela Cruz, PhD^1^; Rose Constantino^2^; Pearl Cuevas, PhD, MAN, BSN, RN^2^**

^1^Centro Escolar University, Manila, Philippines

^2^University of Pittsburgh SON, Pittsburgh, PA, USA

**Background:** The Filipino older person poses a great economic challenge as their productivity declines There is an urgent demand for commitment, action, and research to assess and respond to the various needs of this population group. The Commission on Philippine Human Rights in 2014 documented a total of 760 human rights violation cases involving victim aged 60 and above. There is a range of 3.2 to 27.5% elder abuse reported by the general public, of which the adult children are the main perpetrators. 

**Purpose:** The purpose of this research was to assess the HEARTS of older persons in the Philippines. An Acronym used for H-Health, EA- Experience of Abuse, R-Resilience, T-Treatment, and S-Safety. The researchers aimed to identify interventions that will enhance health and experience elder abuse in an urban city in the Philippines. 

**Methods:** The study explored the HEARTS of older persons through a descriptive survey quantitative research using the PROMIS (Patient-Reported Outcomes Measurement Information System) Questionnaire that determined the health status and the EASI (Elder Abuse Suspicion Index) Questionnaire that determined the experience of abuse. Both standardized questionnaires are translated and back-translated into Filipino. The research question was whether experiencing multiple major health events diminishes rates of resilience. 

**Results:** The scores of health and abuse correlation matrix show a correlation coefficient of 0.461 between the abuse score and the overall health score. The correlation coefficient for physical health is −0.307, for anxiety, is −0.324, for depression is -0.429 and for fatigue is -0.429. The p-value of physical health is 0.006, anxiety is 0.003, depression and fatigue are 0. The over-all p-value score is 0 and is significant at the set 0.05 level for the 2-tailed test. Findings showed the experience of abuse and health on the other hand yield significant p-values of less than 0.05. All of the scores for health has a negative moderate correlation. 

**Conclusions:** Abuse in older persons affects their health. An older person who is not experiencing abuse is most likely healthy. The study gave evidence that Filipino older persons are doing all that they can to get their basic necessities and medical needs for themselves. However, the study shows that research needs to be done to examine the welfare of Filipino older persons to safeguard their health and well-being.

**Symposium 3: Abstract 4**

The Treatment Of Social Networks & Safety Status Of Filipino Older Persons Experiencing Elder Abuse

**Pearl Cuevas, PhD, MAN, BSN, RN^1^; Rose Constantino^2^; Elvira L. Urgel^1^**

^1^Centro Escolar University, Manila, Philippines 

^2^University of Pittsburgh School of Nur, Pittsburgh, PA, USA

To Filipinos, the family is the center of the social structure. Providing care for the older person remains a moral obligation for families in the Philippines. However, with the continuous change in the economy, there are many families in the Philippines who at times can no longer provide the necessities making them incapable of fulfilling their duty to support the older person under their care.

**Purpose:** The purpose of this research was to assess the HEARTS of older persons in the Philippines. An Acronym used for H-Health, EA- Experience of Abuse, R-Resilience, T-Treatment, and S-Safety. The treatment of social networks and their safety were assessed in this study. Results of this research could benefit older persons towards the comprehensive, person-centered, efficient, and effective delivery of nursing care that is responsive to their needs.

**Methods:** The study explored the HEARTS of older persons and used an Interview schedule with open-ended questions that determined the treatment and safety of older persons. The expert made a 10 item open-ended research questionnaire for the interview schedule. These were tape-recorded and transcribed verbatim. The interview schedule generated rich information from the participants by using open-ended questions. Colaizzi’s distinctive seven-step process provides a rigorous analysis, with each step staying close to the data.

**Results:** We generated findings that resulted in themes for active and frail older persons. The major themes are social network and self. The first theme is a social network that includes three (3) subthemes, 1) support from significant others, 2) unhealthy communication, and 3) unfair treatment. The second theme is self which also includes three (3) subthemes, 1) self-diversion, 2) ineffective coping and 3) effective coping. For the active older persons, the major themes are self and social network including three (3) subthemes. We will be presenting in detail each theme, subthemes, and mini-themes for clarity and transparency including the concepts autonmy or respect, justice or fairness, and blessing or high regard. 

**Conclusions:** The study gave evidence that older persons use self- help activities in order to relieve the pains of abuse through interactions/communication with other people. . They are vigilant with their surrounding because they fear for their safety.

**Symposium 3: Abstract 5**

Resilience Interventions for Frail Older Persons In The Experience Of Abuse

**Elvira L. Urgel, PhD, RN^1^; Rose Constantino, PhD, RN^2^**

^1^Centro Escolar University, Manila, Philippines 

^2^University of Pittsburgh School of Nursing, Pittsburgh, PA, USA

**Background:** Assessing the HEARTS of older persons in the Philippines is an important aspect of nursing care. An acronym for H-Health, EA-Experience of Abuse, R-Resilience, T-Treatment, and S-Safety is a comprehensive screening and assessment process that is essential in developing evidence-based interventions. 

**Purpose:** The study also aimed to identify interventions that enhance resilience in older persons experiencing depression, anger, anxiety, and elder abuse. 

**Methods:** The study used a mixed methods approach in the Sequential Transformative design, wherein the participants were allowed to respond to objective standardized questionnaires. The total number of older person participants (N = 80) is further divided into two groups: 40 active older persons and 40 frail older persons who were all free from co-morbid conditions that may result in memory loss or cognitive impairment. The interview schedule was translated in Filipino with open-ended questions that determined treatment and safety of older persons. A registered psychologist was on standby during the interview for any concerns with the mental being of the respondents. A tape recorder was used for the interviews and transcribed in verbatim. 

**Results:** Results showed the resilience interventions of frail older persons in the experience of abuse. These are categorized into major themes: self-reliance, social networking. This statement, “My children are always here to support me and remind me not to overthink. What they do is take me out on a family date“. This person is thankful for her family who looks after her. Those who relied on social networks or significant others were grateful for the communication “When my friends know that I’m bothered they would talk to me. They will listen to me then they will give advice to not stress over my problems and before I think about them, think about my self first.”. Respect: “They keep quiet when I get mad. They just follow my instructions.”. Obedience: “My children don’t rebel against me. 

**Conclusions:** Interventions emphasizing optimism and positive emotions may be particularly effective in building resilience. The study gave evidence that older persons seek the help of social networks in times of stresses or problems. A novel finding is that Filipinos take pity on older persons, therefore, their neighbors look after them. The older persons still manage to survive on a daily basis because of the good treatment of their community. 

